# Crystal Structure of the Tetrameric Fibrinogen-like Recognition Domain of Fibrinogen C Domain Containing 1 (FIBCD1) Protein*

**DOI:** 10.1074/jbc.M113.520577

**Published:** 2013-11-28

**Authors:** Annette K. Shrive, Jesper B. Moeller, Ian Burns, Jenny M. Paterson, Amy J. Shaw, Anders Schlosser, Grith L. Sorensen, Trevor J. Greenhough, Uffe Holmskov

**Affiliations:** From the ‡ Research Institute of Science and Technology in Medicine, School of Life Sciences, Keele University, Staffordshire ST5 5BG, United Kingdom and; the § Department of Cardiovascular and Renal Research, Institute of Molecular Medicine, University of Southern Denmark, DK-5000 Odense, Denmark

**Keywords:** Crystal Structure, Ligand-binding Protein, Pattern Recognition Receptor, Receptor Structure-Function, Structural Biology, FIBCD1, Acetyl Binding, Chitin Receptor, Fibrinogen-like Domain

## Abstract

The high resolution crystal structures of a recombinant fragment of the C-terminal fibrinogen-like recognition domain of FIBCD1, a vertebrate receptor that binds chitin, have been determined. The overall tetrameric structure shows similarity in structure and aggregation to the horseshoe crab innate immune protein tachylectin 5A. The high affinity ligand *N*-acetylmannosamine (ManNAc) binds in the S1 site, predominantly via the acetyl group with the oxygen and acetamide nitrogen hydrogen-bonded to the protein and the methyl group inserted into a hydrophobic pocket. The binding of the ManNAc pyranose ring differs markedly between the two independent subunits, but in all structures the binding of the *N*-acetyl group is conserved. In the native structure, a crystal contact results in one of the independent protomers binding the first GlcNAc of the Asn^340^ *N*-linked glycan on the other independent protomer. In the ligand-bound structure this GlcNAc is replaced by the higher affinity ligand ManNAc. In addition, a sulfate ion has been modeled into the electron density at a location similar to the S3 binding site in L-ficolin, whereas in the native structure an acetate ion has been placed in the S1 *N*-acetyl binding site, and a sulfate ion has been placed adjacent to this site. These ion binding sites are ideally placed to receive the *N*-acetyl and sulfate groups of sulfated GalNAc residues of glycosaminoglycans such as chondroitin and dermatan sulfate. Together, these structures give insight into important determinants of ligand selectivity, demonstrating versatility in recognition and binding while maintaining conservation in *N*-acetyl and calcium binding.

## Teaser

**Background:** FIBCD1 is a tetrameric plasma membrane protein that uses a fibrinogen-like recognition domain (FReD) for pattern recognition of acetyl groups on chitin.

**Results:** The x-ray structure of the FIBCD1 FReD reveals how FIBCD1 binds acetylated and sulfated molecules.

**Conclusion:** FReD domains combine versatility with conservation to recognize their targets.

**Significance:** The structure suggests how FIBCD1 binds acetylated pathogen-associated molecular patterns (PAMPS) and endogenous glycosaminoglycans.

## Introduction

Fibrinogen-like recognition domain containing 1 (FIBCD1)^3^ is a recently discovered vertebrate acetyl group recognition receptor that binds chitin (1). FIBCD1 forms tetramers in the plasma membrane, and each of the chains of the homotetrameric protein consists of a short cytoplasmic tail, a trans-membrane helix, and an ectodomain containing a coiled-coil region, a polycationic region, and a C-terminal fibrinogen-like recognition domain (FReD).

FIBCD1 is expressed mainly apically on enterocytes and on airway epithelial cells, but also on epithelial cells lining the salivary ducts. FIBCD1 mediates endocytosis of its bound ligand which is released to the surroundings after degradation, with FIBCD1 being recycled to the plasma membrane. Two potential phosphorylation sites in the cytoplasmic part of FIBCD1 suggest that FIBCD1 also may be a signaling protein.

**FIGURE 1.**
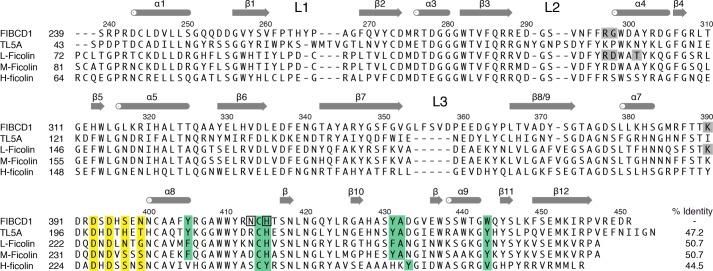
**Alignment and sequence homology (identity) of the fibrinogen-like domains of FIBCD1, TL5A, L-ficolin, M-ficolin, and H-ficolin based on structural superposition.** Sequence numbers and secondary structure elements on the *top* refer to the FIBCD1 sequence with the numbering of the helices and strands based on the secondary structure elements assigned in TL5A and L-ficolin. The loops L1, L2, and L3 (see “Results”) are indicated. The S1 and calcium binding site residues are highlighted in *green* (S1) and *yellow* respectively, with the S3 binding site highlighted in *gray*. Residues that bind the additional sulfate in proximity to S1 are *boxed*.

The FIBCD1 gene is localized on chromosome 9q34.1 in close proximity to the genes encoding L- and M-ficolin (2). The FReD of FIBCD1 shows high homology to the vertebrate innate immune proteins L-ficolin and M-ficolin and to the horseshoe crab protein tachylectin 5A (TL5A) that all bind acetyl groups through the fibrinogen-related domain (Fig. 1). FIBCD1 specifically binds acetylated components including chitin, but fails to bind lipopolysaccharides (LPS), lipoteichoic acid, mannan, or peptidoglycan (1), the last consisting of GlcNAc and MurNAc residues arranged in a structure similar to that of the (GlcNAc)*_n_* structure of chitin. This binding is in contrast to L-ficolin whose known ligands, in addition to acetyl groups (3), include lipoteichoic acid and β-1,3-glucan (4), and to TL5A, which recognizes the *O*-antigen of LPS (5). An extended binding surface that incorporates four binding sites designated S1–S4, has been identified in L-ficolin, with various carbohydrate and noncarbohydrate ligands binding to sites S2–S4 (6). In contrast to TL5A (7) and M-ficolin (8), which specifically bind *N*-acetyl groups in site S1, acetylated ligands bind to L-ficolin in either S2 or S3 depending on the nature of the ligand (6).

The high homology to the ficolins, which are well characterized pattern recognition molecules that play important roles in innate immunity, and the location at the apical part of mucosal epithelial cells suggest that FIBCD1 plays an important role in innate immunity. The oligomeric state of FIBCD1 supports this, as oligomerization allows structural arrangement so that an appropriate number of binding sites match the spatial arrangement of microbial molecular patterns, leaving endogenous ligands unbound due to alternative spacing. A role in homeostasis cannot be ruled out as many repeating acetylated components are present in, for example, mucins on mucosal surfaces.

FIBCD1 is the first characterized plasma membrane protein that exploits a FReD as ligand binding domain. In contrast to the well characterized ficolins that form homotrimers, FIBCD1 is thought to form homotetramers. We here report the refined three-dimensional structures of the FReD domain of FIBCD1 with and without bound ligand. We show that the FReD of FIBCD1 indeed forms homotetramers of protomers with high homology to the soluble horseshoe crab protein tachylectin 5A. The results reveal not only the structural basis of both the tetramerization of the FIBCD1 FReDs and acetyl group-specific ligand binding through the S1 site, but also potential binding sites for sulfated ligands including glycosaminoglycans such as chondroitin and dermatan sulfate.

## EXPERIMENTAL PROCEDURES

### 

#### 

##### Cloning, Expression, and Purification of the Fibrinogen-related Domain of FIBCD1

The DNA segment corresponding to the fibrinogen-related domain of human FIBCD1 (residues 236–461) was cloned into the pNT-Bac vector (9) and expressed in insect cells as described previously (1). Purification of the fibrinogen-related domain of FIBCD1 was achieved by affinity chromatography using acetylated Toyopearl AF-Amino-650M resin (Tosoh) essentially as described previously (1), followed by ion-exchange chromatography using a Resource Q ion-exchange column (GE Healthcare). In brief, eluates containing affinity-purified recombinant FIBCD1 were pooled and diluted 1:20 in TE buffer (10 mm Tris, 5 mm EDTA, pH 7.4) before being applied onto the column. The column was washed with 10 ml of TE buffer followed by 20 ml of 10 mm Tris, pH 7.5, and elution was performed by a two-step gradient of NaCl (0–200-1000 mm). The fractions containing recombinant FIBCD1 were analyzed by SDS-PAGE/Coomassie staining and finally dialyzed against TBS (10 mm Tris, 140 mm NaCl, 0.02% NaN_3_, pH 7.4).

##### Crystallization and Data Collection

Recombinant FIBCD1 was concentrated, using Amicon® Ultra concentrators (Millipore), to 8 mg/ml in 10 mm Tris, 140 mm NaCl, 10 mm CaCl_2_, 0.02% NaN_3_, pH 7.5, for crystallization. Native crystals of the fibrinogen domain (residues 236–461) were grown in sitting drops consisting of an equal volume (1.5–2 μl) of protein solution and precipitant buffer of 1.6–1.7 m (NH_4_)_2_SO_4_, 7–10% dioxane, 0.1 m MES, pH 6.5. Crystals were prepared for cryocooling using glycerol in precipitant buffer with the addition of 10 mm CaCl_2_. Successive addition of 2-μl aliquots of increasing concentrations (5–25%) of glycerol cryobuffer were added to the well, followed by addition of a further 2-μl aliquot of 25% glycerol cryobuffer and an exchange of ∼10 μl of the resulting buffer with 25% glycerol cryobuffer. Ligand was introduced into the crystal by the addition of 10 mm ManNAc to the cryobuffer. Data were collected, from a single crystal in each case, on an ADSC Quantum 4R CCD detector at Daresbury SRS (14.1) and an ADSC Q315r at Diamond Light Source (I04). Integrated intensities were processed using MOSFLM (10) and CCP4 programs (11). Data collection and processing statistics are given in Table 1.

**TABLE 1:** **Data collection and processing** Figures in parentheses refer to the highest resolution bin.

**Data collection**	**Native**	**ManNAc bound**
Synchrotron station	SRS 14.1	DLS I04
Wavelength (Å)	1.488	0.9745
Space group	P4	P4
Cell dimensions	*a* = *b* = 118.56 Å, *c* = 44.25 Å	*a* = *b* = 119.54 Å, *c* = 44.26 Å
Resolution range (Å)	41.9–2.0 (2.11–2.00)	53.5–2.1 (2.21–2.10)
Observations	130,094 (16,153)	156,110 (23,101)
Unique reflections	41,125 (5,672)	36,910 (5,361)
Completeness (%)	97.8 (93.3)	99.8 (100.0)
*R*_merge_*^a^*	0.066 (0.214)	0.069 (0.174)
*I*/σ(*I*)	8.0 (2.9)	6.1 (4.2)

**Refinement**		
Protein atoms	3,520	3,531
Residues chain A	239–457	239–458
Residues chain B	239–457	239–457
Water molecules	297	321
Other molecules		
Subunit	A B	A B
Calcium ions	1 1	1 1
Sulfate ions	2 1	1 1
Acetate ions	1	
GlcNAc	1	1
Glycerol	1	
ManNAc ligand		1 1
*R*_work_*^b^* (%)	18.3	18.7
*R*_free_*^c^* (%)	20.9	21.4
r.m.s.d.*^d^* bond length (Å)	0.005	0.006
r.m.s.d. bond angle (°)	1.32	1.30
Average *B*-values (Å^2^)		
Protein	20.2	16.9
Water	32.4	28.8
Other hetero-atoms	40.7	34.1
PDB ID	4M7H	4M7F
**Ramachandran plot values*^e^* (%)**		
Favored	93.3	93.5
Allowed	6.7	6.5
Outliers	0.0	0.0

*^a^ R*_merge_ = Σ*_h_*Σ*_j_*|*I_h_*_,_*_j_* − *I_h_*|/Σ*_h_*Σ*_j_*|I*_h_*_,_*_j_*|, where I*_h_*_,_*_j_* is the *j*th observation of reflection *h* and *Ih* is the mean of the *j* measurements of reflection *h*.

*^b^ R*_work_ = Σ*_h_*‖*F*_oh_| − |*F*_ch_‖ /Σ*_h_* |*F*_oh_| where *F*_oh_ and *F*_ch_ are the observed and calculated structure factor amplitudes, respectively, for the reflection *h*.

*^c^ R*_free_ is equivalent to *R*_work_ for a randomly selected subset (5%) of reflections not used in the refinement.

*^d^* r.m.s.d., root mean square deviation.

*^e^* Defined according to Molprobity.

##### Structure Solution and Refinement

The native FIBCD1 structure was solved by molecular replacement with AMoRe (12) using the homologous tachylectin 5A structure (Protein Data Bank ID code 1JC9) as a search model. The refined native structure was then used as a starting model for the ligand-bound structure. As the crystals were isomorphous, molecular replacement was not necessary for the ligand structure. Model building of the structures was carried out using maximum likelihood refinement with CNS (13) and alternated with rounds of manual model building with O (14). Topology and parameter files for ligand were obtained from the HIC-Up server (15). Refinement statistics are given in Table 1, and the quality of the final structures was verified by MolProbity (16). The structures have >93% residues in favored regions of the Ramachandran plot with no outliers. Residues 239–457/8 of FIBCD1 have been fitted into the electron density. The coordinates and structure factors for native (4M7H) and ManNAc-bound (4M7F) FIBCD1 have been deposited with the Protein Data Bank. Molecular figures were generated using MOLSCRIPT (17) and the PyMOL Molecular Graphics System Version 1.4 (Schrödinger, LLC, 2011).

## RESULTS

A single species of the expressed and purified FIBCD1 segment corresponding to residues 236–461 was produced with an average mass of 27.3 with a spread of ± 0.8 kDa as determined by MALDI-MS. The mass was greater than the calculated mass (25.9 kDa) based on the amino acid sequence, probably due to glycosylation (see below) during biosynthesis (2).

### 

#### 

##### Overall Structure

The structure of the recombinant glycosylated FReD of FIBCD1 was solved by molecular replacement using the homologous TL5A structure (7) as a search model and subsequently refined to a resolution of 2.0 Å for the native fragment and 2.1 Å for the crystals soaked in ManNAc (Table 1).

**FIGURE 2.**
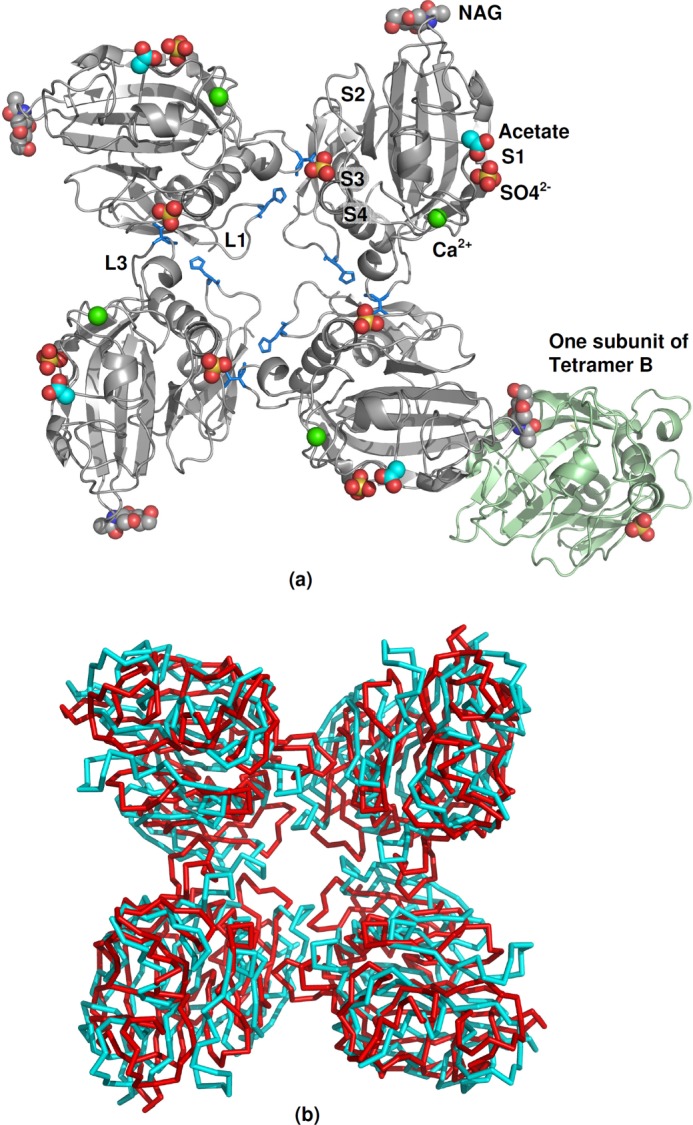
**Homotetrameric structure of the recognition domains of FIBCD1.** *a*, subunit A tetrameric native structure of FIBCD1 illustrating the crystal contact, mediated through the *N*-linked glycan, with the subunit B tetramer (one protomer shown in *green*). The four binding sites S1–S4 are labeled. The key amino acids His^264^ and Val^357^ at the protomer-protomer interface in loops L1 and L2, respectively, are shown as *stick models. b*, overlay of the FIBCD1 and TL5A tetramers showing the relative orientation of the protomers within the tetrameric molecule.

The crystal structure contains two independent tetramers (one composed of subunits A, the other of subunits B) in the unit cell (Fig. 2). Each of these tetramers has 4-fold molecular symmetry, tetramer A being positioned on the crystallographic 4-fold axis which is parallel to *z* (*c*) at *x* = 0, *y* = 0 and tetramer B on the 4-fold axis which is parallel to *z* at *x* = 1/2, *y* = 1/2. Residues 239–457 are observed in the electron density for both subunits. There is clear evidence for glycosylation at Asn^340^, the *N*-linked GlcNAc in one independent subunit (subunit A) being clearly defined due to crystal contacts whereas in subunit B the electron density does not allow linked carbohydrate to be modeled with confidence.

There are extensive interactions between neighboring protomers in the biologically relevant tetramer, involving the loop L1 (Fig. 1), which connects strands β1 and β2 (residues ∼261–268), and loop L3 (352–363) which includes a helical region (α6) in L-ficolin (6). Loop L1 in each of the four protomers in the tetramer contacts the same loop in each of the two neighboring protomers, forming the major contact interface close to the 4-fold axis. His^264^ inserts into a pocket in the neighboring L1 (Fig. 2), forming hydrogen bonds with the main chain carbonyl of Ala^267^ (ND1-O 2.80Å) and with Ser^259^ OG (NE2-OG 2.72Å), whereas there is a hydrophobic interaction between Thr^263^ CG2 and Phe^261^. In loop L3 the side chain of Val^357^ extends into a hydrophobic pocket in the β5-α5 region of the neighboring protomer, with Val^357^ encircled by the side chains of Leu^309^ and Leu^315^ and the main chain of residues 305–309 and 313–315.

In both native and ligand-bound structures, electron density in the region corresponding to the acetyl binding site (S3) in L-ficolin has been modeled as a sulfate ion, one of the S3 sulfate oxygens interacting with Arg^297^NE (3.1Å), the main chain nitrogen of Gly^298^ (2.7 Å) and a water molecule. A second sulfate oxygen also interacts with Arg^297^NE although the distance is slightly greater, and with Lys^390^NZ.

##### Calcium Binding

A calcium ion is located in each protomer in sites homologous to the calcium site in TL5A and the ficolins (Fig. 2), coordinated here by Asp^393^ (×2), Asp^395^, the main chain carbonyls of Ser^397^ and Asn^399^, and two water molecules. Each calcium ion is 7-coordinated with Asp^395^ and one water forming the vertices of a pentagonal bipyramid and the remainder forming the pentagonal base. The average Ca-O bond distance in each of the two subunits in each of the two structures agrees with the characteristic value of 2.4 Å for Ca^2+^ binding sites in proteins (18). The 400–405 helix α8 flanks the Ca^2+^ binding site and connects the metal binding site to the acetyl group recognition site via the Cys^401^-Cys^414^ disulfide with a *cis*-peptide bond between Asn^413^ and Cys^414^.

##### Native Structure

**FIGURE 3.**
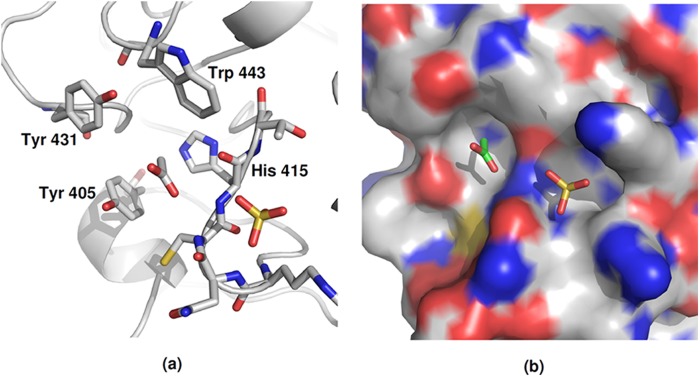
**Acetyl binding site S1 in each protomer of the subunit A tetramer of the native FIBCD1 structure.** The acetate and sulfate ions located in and in proximity to the S1 acetyl binding pocket are shown. *a*, key interacting amino acids. *b*, charged surface representation of the extended S1 site including the acetyl binding pocket and the adjacent pocket which accommodates a sulfate ion.

**FIGURE 4.**
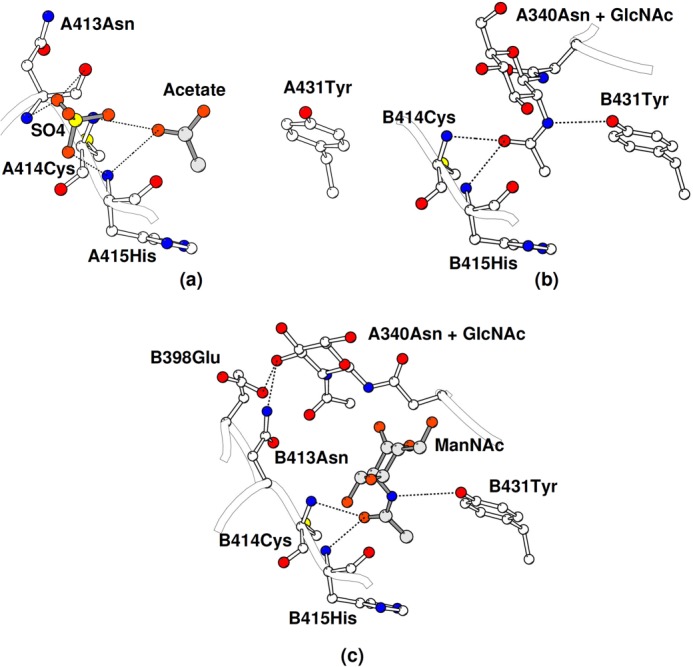
**Acetyl binding site S1 in FIBCD1 showing the key amino acids and interactions between bound ligand and protein.** *a*, native FIBCD1 subunit A showing the acetate and sulfate ions. *b*, native FIBCD1 subunit B showing the Asn^340^ glycan GlcNAc from the subunit A tetramer inserted in to the acetyl binding pocket. *c*, subunit B of the ManNAc-bound structure showing the bound ManNAc and the displaced subunit A glycan.

Electron density in the acetyl position of the ligand binding site (as seen in TL5A and designated S1 in ficolins) is present in both subunits of the native FIBCD1 crystal structure. In subunit A this density corresponds closely to an acetate ion, and this has been fitted. In close proximity to this acetate in the S1 binding site of subunit A, a sulfate ion has been modeled into a large piece of electron density (Figs. 3 and 4*a*). This sulfate ion interacts with the protein main chain through O2-His^415^N (3.2 Å), and through O4-Asn^413^N and O4-Asn^413^O at 3.0 and 3.1, Å respectively.

**FIGURE 5.**
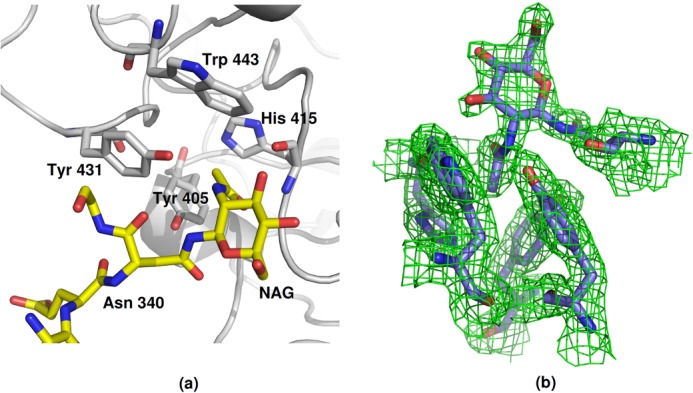
**Acetyl binding site S1 in each protomer of the subunit B tetramer of the native FIBCD1 structure.** The Asn^340^ glycan GlcNAc from the subunit A tetramer inserts in to the acetyl binding pocket S1 of subunit B. *a*, structure of the binding site and the bound glycan. *b*, 2*F_o_* − *F_c_* electron density contoured at 2σ.

In the other independent subunit (subunit B) in the native structure, a crystal contact results in the Asn^340^ *N*-linked GlcNAc from subunit A being bound in the subunit B ligand binding site S1 (Figs. 4*b* and 5). There are no substantial differences in conformation between the two independent subunit ligand binding sites except that in subunit B the conserved Tyr^431^ moves in compared with subunit A, where the closest approach of Tyr^431^OH to the isolated acetate ion is 4.6 Å to an acetate oxygen, to interact with the N of the *N*-acetyl group of the glycan GlcNAc (Tyr^431^OH-acetamide N 3.0A). The acetyl oxygen is bound by two adjacent main chain nitrogens from Cys^414^ and His^415^, the latter being maintained in this orientation through the *cis*-conformation of Cys^414^. The *N*-acetyl methyl group sits in a conserved hydrophobic and aromatic pocket surrounded by Tyr^405^, His^415^, Tyr^431^, and Trp^443^, contact distances with these residues ranging from 3.67 Å (Tyr^405^CZ) to 3.93 Å (Tyr^431^CE2) (Figs. 4*b* and 5). Although there is evidence of electron density for the second, linked GlcNAc of the bound glycan, it is ill defined and of insufficient quality to allow fitting.

##### ManNAc-bound Structure

**FIGURE 6.**
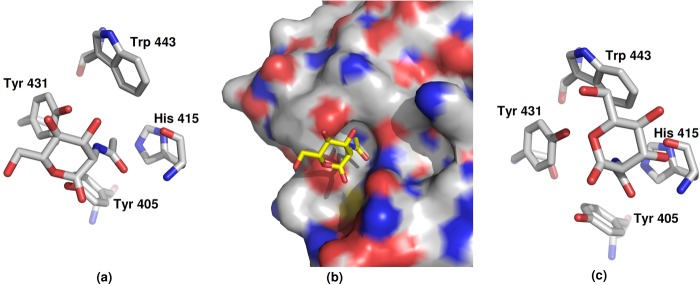
**Acetyl binding site S1 in the ManNAc-bound FIBCD1 structure.** *a* and *b*, binding site in each protomer of the subunit A tetramer. *c*, binding site in each protomer of the subunit B tetramer where the *N*-linked GlcNAc from the subunit A tetramer in the native structure is displaced by ManNAc.

**FIGURE 7.**
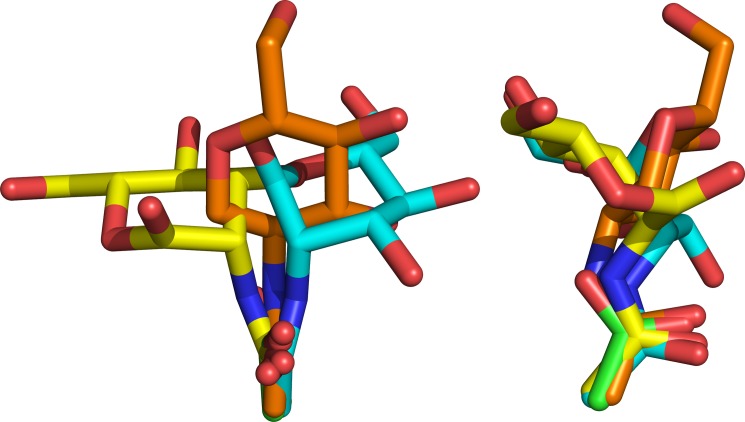
**Orthogonal views of the overlaid bound ligands in the FIBCD1 S1 acetyl binding site generated by superposing (least squares fit of the main chain atoms) subunits A and B in both the ManNAc-bound structure and the native structure.** Ligands shown are ManNAc in the subunit A tetramer of the ManNAc-bound structure (*yellow*), the *N*-linked glycan GlcNAc from the subunit A tetramer bound in the native subunit B tetramer (*orange*), the acetate ion in the subunit A tetramer of the native structure (*green*), and ManNAc in the subunit B tetramer of the ManNAc bound structure (*cyan*).

In the ManNAc ligand-bound structure there are major differences, due to the crystal contacts, in the orientation of the ligand and its interactions in the two independent subunits (Figs. 4 and 6). Nevertheless, the position, orientation, and interactions of the *N*-acetyl group are conserved (Fig. 7). In subunit A, the acetate, but not the sulfate ion, in the native structure has been displaced by ManNAc whereas in subunit B the GlcNAc of the glycan is displaced from the binding site where it is replaced by ManNAc. This displacement is accompanied by a significant change in conformation of Asn^340^ in subunit A which holds the *N*-linked glycan. The ManNAc *N*-acetyl group in both subunits interacts with Tyr^431^ and the main chain nitrogens of Cys^414^ and His^415^, with the methyl group inserting into the hydrophobic pocket. In subunit A Tyr^431^ moves toward the ligand to form a hydrogen bond (3.1 Å) between the *N*-acetyl nitrogen and the Tyr^431^ hydroxyl. The major difference between the ManNAc in the two different subunits is a rotation of approximately 60° of the pyranose ring about the acetyl C-N bond. In subunit A this results in a close (2.3 Å) contact between ManNAc O1′ and the main chain carbonyl of Asn^413^, with the ManNAc O1′ and O6′ hydroxyls forming water-mediated contacts with the Tyr^405^ hydroxyl. In subunit B the displaced GlcNAc moves out of the ligand binding site, ManNAc O3′ interacting with the main chain carbonyl of His^415^ at 2.77 Å with an unusually long 3.5 Å Tyr431OH-acetamide N interaction. The O3′ hydroxyl of the displaced glycan GlcNAc interacts with the side chains of Glu^398^ and Asn^413^ at the protein surface. There is also a clearer indication than in the native structure of electron density in the region of GlcNAc O4′ for the first part of the adjoining GlcNAc of the glycan. There is no evidence that residue Lys^381^ (equivalent to the ligand binding Arg^186^ in TL5A; see Fig. 1) interacts with either the bound ManNAc or the bound glycan GlcNAc in the native structure or with the sulfate ion close to the native acetate site.

## DISCUSSION

We have determined the three-dimensional structure of the fibrinogen-like recognition domain of human FIBCD1. The FReD-1 domain of FIBCD1 has an overall protomer topology that is similar to that of TL5A and the ficolins, forming a tetramer in agreement with the proposed association to form noncovalent tetramers (2) as observed for TL5A (7). Although the tetrameric arrangements of FIBCD1 and TL5A look similar, there is a rearrangement of the protomers within the tetramer with the FIBCD1 subunit rotated by ∼23° about an axis parallel to the tetramer axis (*z* axis) with respect to the TL5A protomer (see Fig. 2). This appears to be the result of the sequence differences (insertions/deletions) between loops L1 and L3 in FIBCD1 and TL5A (Fig. 1). In TL5A the two loops, which, unlike FIBCD1, include short α-helical structures, interact with each other across the interprotomer interface, dominated by the interaction of Trp^161^ at the start of L3 with Arg^64^, Thr^75^, and Asn^77^ in the β2-L1-β3 region of the neighboring protomer (7). In FIBCD1, however, the major contact interface close to the 4-fold axis is formed by L1-L1 interactions. In addition, Val^357^ in FIBCD1 loop L3 extends into a hydrophobic pocket in the β4-β5 region of the neighboring protomer, the equivalent interaction in TL5A being a side chain stacking of Tyr^167^ (L2) and Arg^129^ (β5).

Thus, as expected from sequence homology, the overall protomer fold of the FReD-1 domain of FIBCD1 is the same as that of TL5A and the ficolins, whereas the tetramer itself differs due to sequence differences at the subunit-subunit interface. This is reminiscent of the human innate immune pentraxins SAP and CRP, where the protomer fold is closely similar, but again the orientation of the protomers in the biological pentamer differs (19, 20), by approximately 15°. In both cases structure solution by molecular replacement requires a monomer model to be successful (21).

Within each protomer a calcium ion is located in sites homologous to the calcium site in TL5A and the ficolins, with equivalent residues and water coordinating the calcium ion. This site is connected to the acetyl group recognition site S1 via the Cys^401^-Cys^414^ disulfide, equivalent to the Cys^206^-Cys^219^ disulfide bridge in TL5A.

The Asn^413^-Cys^414^ *cis*-peptide bond is equivalent to that between Arg^218^ and Cys^219^ in TL5A. Both position backbone NH groups (Cys^414^ and Cys^415^ here; Cys^219^ in TL5A) to interact directly with the bound acetyl group of the ligand thus contributing substantially to the acetyl group specificity (7) (see below). This *cis*-peptide bond also corresponds to the pH-dependent *cis/trans* bond seen for M-ficolin (8), perhaps corresponding to a regulatory mechanism for ligand binding, the S1 site being disrupted by a transition of the peptide bond to *trans* at acidic pH.

The origin of the acetate ion in the ligand binding site of subunit A of the native structure is unclear (Fig. 3). Although acetate has not been used in the protein buffer or crystallization conditions, sodium acetate is used in the purification procedure and may have been bound at this time. The sulfate ions, in close proximity to the S1 acetate in subunit A and at the S3 site, however, could have arisen from the ammonium sulfate or MES present in the crystallization condition (see Fig. 3). Electron density in close proximity to O3′ of the bound glycan may correspond to the second GlcNAc of the glycan, expected at the neighboring O4′.

Binding of the *N*-acetyl group is conserved throughout the structures, the acetyl nitrogen interacting with the conserved Tyr^431^ and the oxygen with two adjacent main chain nitrogens from Cys^414^ and His^415^, both positioned by the *cis*-conformation of Cys (Fig. 4). The hydrophobic and aromatic pocket which holds the *N*-acetyl methyl group is formed by residues Tyr^405^, His^415^, Tyr^431^, and Trp^443^, with Ala^432^ at the base of the pocket (Fig. 6). All of these residues are conserved in FIBCD1, TL5A, and L- and M-ficolin except for Trp^443^ which is Tyr in TL5A and in L- and M-ficolin (Fig. 1). Although the distance from the *N*-acetyl C8 to Ala^432^ CB is somewhat long, for example 3.8 Å in the GlcNAc-bound structure, no other amino acid would appropriately complete the pocket without spatially preventing entry of C8. Trp^443^ is clearly and unambiguously defined in the GlcNAc (subunit B, native structure) and ManNAc (subunits A and B ManNAc-bound structure)-bound subunits, whereas in the absence of a bound carbohydrate (subunit A, native structure) the density is less well defined, suggesting that this residue has some freedom of movement which is stabilized by interaction with bound ligand.

In addition to the S1 ligand-binding site in TL5A and the ficolins, L-ficolin is reported to contain three additional binding sites (S2–S4) surrounding a cleft to form an extended ligand binding surface. There is generally a conservation of the S1 pocket in FIBCD1, TL5A, and H- and M-ficolin. TL5A, H-, and M-ficolin bind acetylated structures through S1 whereas L-ficolin binds acetylated structures primarily through S3 or S2. The FIBCD1 site equivalent to the acetyl binding site S3 in L-ficolin contains a sulfate ion and the FIBCD1 S3 residues Lys^390^ and Arg^297^ which interact with this sulfate ion are equivalent to those in L-ficolin S3, Lys^221^ and Arg^132^ (6).

An overlay of the FIBCD1 protomers (main chain) shows that the isolated acetate in the native FIBCD1 structure is essentially in the same position and orientation as the *N*-acetyl group in TL5A and M- and H-ficolins and in the bound ManNAc and (glycan) GlcNAc protomers here, but that the remainder of the ligand, including the orientation of the pyranose ring, differs markedly (Figs. 5–7). This difference is due to the *N*-linked glycan constraints placed on the GlcNAc and the crystal contacts that affect the orientation of the ManNAc in the subunit B tetramer. The unusually long Tyr^431^OH-acetamide N interaction in subunit B of the ManNAc-bound structure, which may arise from the influence of crystal contacts, may indicate that this interaction, at least for ManNAc, is relatively less critical for binding than the remaining binding determinants. The O3′ hydroxyl of the displaced glycan GlcNAc interacts with the side chains of Glu^398^ and Asn^413^ at the protein surface.

In TL5A Arg^186^ makes a key interaction with the O1′ hydroxyl of GlcNAc (7). The density for the equivalent FIBCD1 residue Lys^381^ is very poorly defined in all structures suggesting mobility and either that the side chain is too short to reach the sugar, or that it is not part of the mode of binding of the ligands studied here. In the native acetate-bound site the sulfate adjacent to the S1 site is sufficiently close to Lys^381^ for an interaction to occur, but again none is indicated by the electron density. Perhaps this interaction is of importance for longer ligands, for example natural extended carbohydrate ligands.

The acetate and sulfate that are observed in the “native” subunit (A) (Fig. 3) and the position of the extended density that is attached to the GlcNAc glycan sugar (in subunit B) suggest that the S1 binding site in FIBCD1 may well be extended with an ability to bind a variety of ligands in a variety of orientations. The ability to bind both GlcNAc and ManNAc, despite the differing mannose/glucose stereochemistry at the C2 position, is indicative of this flexibility and of the primary requirement for the *N*-acetyl group. It is worthy of note that the S1 site in L-ficolin may also have an extended character and that it too accepts a sugar of a crystal contact glycan, although for L-ficolin a mannose has been assigned to the electron density in the pocket rather than the GlcNAc observed here (6). In L-ficolin the first and second GlcNAc residues of this neighboring oligosaccharide bind to the edge of the S1 site, but on the opposite side of the pocket to the sulfate ion observed here.

Soaking experiments have been carried out to investigate chitobiose binding to FIBCD1, but current electron density maps do not clearly define the bound ligand (data not shown). This suggests that ManNAc, which readily displaces both the acetate and the glycan from the binding site, is a higher affinity FIBCD1 ligand than chitobiose. It may be that chitin binding involves a number of β1–4 GlcNAc residues, interacting not only with the acetyl binding pocket but also the extended GlcNAc (glycan) binding surface adjacent to S1 identified in L-ficolin. Increasing the concentration of low affinity, low occupancy ligands in L-ficolin did not always lead to improvement in quality of electron density maps but rather nonspecific binding to different surface areas (22). FIBCD1, however, has been postulated to be a chitin-binding molecule, and therefore experiments to improve the occupancy of small β1–4 GlcNAc chains in the binding site and to show GlcNAc binding unconstrained by the *N*-link present here, are currently being undertaken.

It will be interesting to see whether Lys^381^ does interact with an extended bound ligand and whether there are further interactions in an extended S1 pocket including either the adjacent GlcNAc binding surface identified in L-ficolin or the site occupied by sulfate in the native FIBCD1 structure. Because FIBCD1 recognizes GlcNAc and GalNAc equally well (2), the proximity of the acetyl and sulfate sites suggests that FIBCD1 may function as a pattern recognition receptor for mucus related sulfated GalNAc residues of glycosaminoglycans such as chondroitin and dermatan sulfate, suggesting a role in mucus homeostasis. Indeed, both the sulfate and the acetyl group of GalNAc 4-sulfate modeled into the extended FIBCD1 S1 site overlie the sulfate and acetate ions observed here (Fig. 3). Structural studies are under way to investigate this previously unreported but potentially significant recognition mode of FIBCD1.

Our structural data indicate that FIBCD1, in line with what is known about the ficolins, plays an important role in innate immunity, acting as a pattern recognition receptor. However, although our data indicate a substantial overlap in ligand binding between FIBCD1 and the ficolins, the FIBCD1 effector mechanisms must be considerably different. After ligand binding the ficolins activate complement through binding of the MASP serine proteases to the collagen regions of the ficolins. No collagen region is found in FIBCD1, and, as FIBCD1 is a membrane protein, the effector mechanism is expected to be endocytosis of bound ligands or signaling. Indeed, we have already shown that FIBCD1 can endocytose acetylated BSA. Future studies will reveal whether FIBCD1 may act as a signaling molecule.

## Footnotes

3 The abbreviations used are:

FIBCD1: fibrinogen C domain containing 1
FReD: fibrinogen-like recognition domain
TL5A: tachylectin 5A.
